# The use of trypan blue to distinguish Pseudo-Seidel sign from lacrimal ductule versus glaucoma drainage device leakage

**DOI:** 10.1016/j.ajoc.2025.102389

**Published:** 2025-07-12

**Authors:** Hamidah Mahmud, Yeabsira Mesfin, Yingna Liu, Yvonne Ou, Jonathan E. Lu

**Affiliations:** a University of California, San Francisco, Department of Ophthalmology, 490 Illinois St., San Francisco, CA, 94158, USA; b University of California, San Francisco, School of Medicine, 533 Parnassus Ave, San Francisco, CA, 94143, USA; c Bay Area Retina Associates, 365 Lennon Lane Suite 250, Walnut Creek, CA, 94598, USA

**Keywords:** Trypan blue, Lacrimal gland, Glaucoma drainage device, Leak, Pseudo-seidel

## Abstract

**Introduction:**

The superotemporal fornix location of the lacrimal gland ductule openings coincides with the typical locations of glaucoma drainage devices; as a result, a conjunctival fistula may look and behave like a lacrimal gland ductule. External morphology and Seidel test are helpful in identifying fluid flow but cannot differentiate the fluid as aqueous humor or tears. We describe a novel technique in differentiating a lacrimal gland ductule from a conjunctival fistula secondary to a glaucoma drainage device.

**Case presentation:**

An 87-year-old female with a superotemporal Ahmed valve in the right eye presented with increased “tearing” over the past year, with concern for repeat tube exposure versus normal secretion from the lacrimal gland ductules. Difficulty in differentiation was further increased due to a regional conjunctival pedicled flap for previous tube exposure. Intra-operatively, trypan blue was injected into the anterior chamber of the right eye. The dye was visualized to track along the tube in the direction of the plate, and brisk flow was then observed at the conjunctival area of ambiguity. This confirmed that fluid leakage was due to a glaucoma drainage device-associated conjunctival fistula. The plate and tube were subsequently removed.

**Conclusions:**

Intraocular injection of trypan blue dye was effective in identifying glaucoma drainage device leakage from tube exposure, and specifically allowing clear differentiation from physiologic lacrimal gland flow. This novel technique successfully differentiated a leaking conjunctival fistula requiring treatment from a benign physiologic finding, in a case where topical fluorescein testing was not sufficient.

## 1 Introduction

Glaucoma drainage devices (GDDs) are typically placed in the superotemporal quadrant of the eye, diverting aqueous fluid from the anterior chamber to the subconjunctival space.^1^ This localization encourages both passive diffusion of aqueous humor from the anterior chamber and creates a filtering bleb that allows nearby tissue to reabsorb the extracted fluid.^2^

Mechanical stress on the overlying conjunctiva can progress to erosion and increases the risk of blebitis and endophthalmitis.^3^ Chronic irritation can contribute towards both erosion and scarring of the conjunctiva, destabilizing the positioning of GDDs and creating anatomic abnormalities such as conjunctival fistulas.^4^^,^^5^ These defects increase the risk of hypotony, infection, and device failure.^5^ Consequently, the immediate diagnosis and management of conjunctival fistulas in patients with GDDs is integral for vision preservation of glaucoma patients.

Current modalities for the detection of conjunctival fistulas are largely effective, but in select scenarios inadequate. The Seidel test, in which fluorescein dye is applied to the ocular surface, is often performed to search for active conjunctival fistulas in patients with GDDs.^6^ The dilution of the dye by leaking aqueous, the “waterfall effect,” confirms the diagnosis of a fistula.^6^ However, given the anatomical proximity of GDDs with the lacrimal gland in the superotemporal quadrant, normal flow from lacrimal gland ductules can be mistaken for a Seidel-positive “waterfall effect,” known as a “pseudo-Seidel” sign.^7^^,^^8^ Both lacrimal gland ductules and conjunctival fistulas in the superotemporal quadrant secrete fluid onto the ocular surface via a conjunctival opening. Fluorescein staining can detect fluid outflow effectively but offers no insight into their source. This limitation of fluorescein dye staining can lead to misdiagnoses and unnecessary treatments for glaucoma patients, including the unnecessary removal or revision of the GDD.

Trypan blue dye is widely used safely during intraocular surgery. Its affinity for the conjunctiva, sclera, and trabecular meshwork allows for precise visualization of ocular tissue.^9^, [10, 11] Following several goniotomies, Laroche et al., demonstrated the implications of such visualization by successfully confirming the patency of the Schlemm's canals and aqueous veins by tracking the drainage of trypan through the trabecular meshwork.^12^ Similarly, Khoo et al., confirmed the presence of aqueous flow through cyclodialysis clefts in patients with recurrent hypotony by injecting trypan blue into their anterior chambers.^13^ Trypan also selectively stains damaged tissue and so is ideal for localizing tissue injury while sparing healthy tissue.^14^ Healey et al., demonstrated how trypan blue prevented excessive cytotoxicity when applying mitomycin C and 5-fluorouracil by clearly delineating the treatment zone.^15^

We hereby report a novel technique of the use of trypan blue to differentiate conjunctival fistulas associated with GDD from physiologic lacrimal fluid secretion.

## 2 Case presentation

**Fig. 1:**
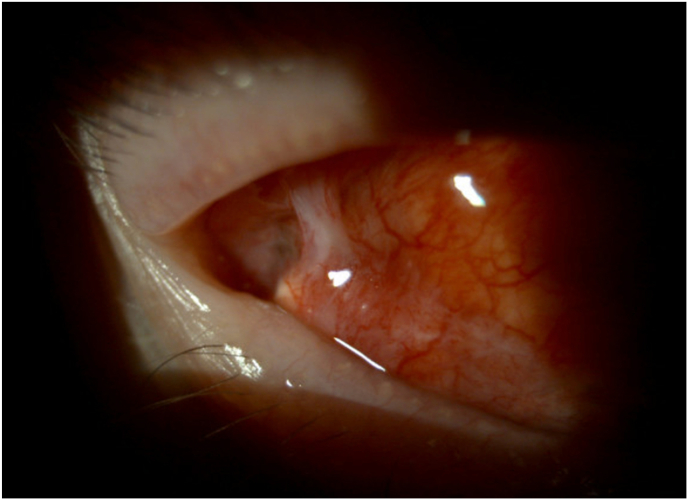
Suspicious area of conjunctival opening in vicinity of glaucoma drainage device. The conjunctival band is a prior pedicled rotational flap to cover previous exposure.

**Fig. 2:**
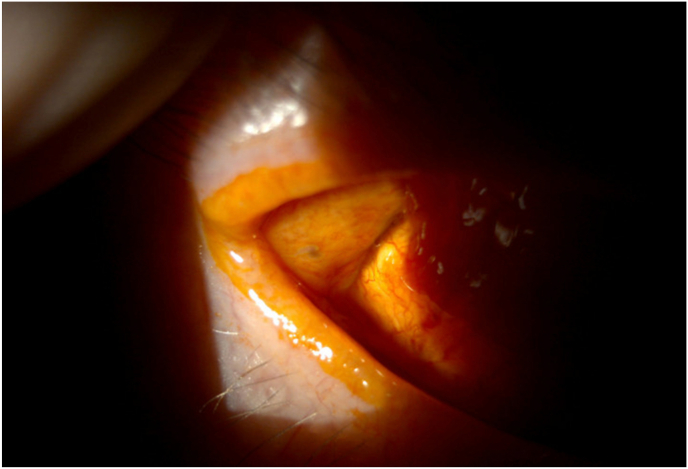
Another conjunctival opening, this one thought more consistent with lacrimal gland ductule.

An 87-year-old woman with a complex past glaucoma history, including initial Ahmed valve implantation for severe mixed mechanism glaucoma in the right eye two years prior to presentation, complicated by tube exposure one year later treated with pedicled conjunctival flap overlay, presented with recurrent tearing, irritation, and a tugging sensation in both eyes. Her glaucoma was secondary to Uveitis-Glaucoma-Hyphema (UGH) syndrome; in addition, the patient suffered from age-related macular degeneration with choroidal neovascularization of the right eye which required recurrent anti-VEGF injections. The original exposure was an asymptomatic 3.7 × 1 mm area over one of the anterior eyelets of the Ahmed plate and adjacent superotemporal scleral exposure of the right eye, which was addressed with tutoplast scleral patch graft re-enforcement and forniceal conjunctival pedicle flap. A year later on initial presentation for this case, her vision was 20/100 right eye and 20/40 left eye. Intraocular pressures (IOP) were 10 and 12 mmHg. Her ocular exam was notable for right sided ptosis and a well-covered superotemporal conjunctival flap with a concerning area of Seidel positivity posteriorly adjacent to the pedicle in proximity to the fornix (Fig. 1, Fig. 2). The Seidel test was performed by instilling 2.5 % fluorescein solution which highlights leaks without requiring the need of a strip or tissue manipulation. The area of possible leak was near the transition of palpebral conjunctiva and bulbar conjunctiva in the fornix, abutting normal physiologic appearing lacrimal gland openings, but with poor visualization in the most suspicious area due to the conjunctival rearrangement. In addition, digital ocular massage did not appear to increase the leak or “waterfall effect.”

Given the ambiguity regarding the presence of a recurrent leak, a diagnostic test with trypan injection in the anterior chamber was recommended in the operating room. In the interim, the patient was prescribed topical antibiotic as infection prophylaxis. The patient was informed of the risks and benefits of the procedure and consented to it.

**Fig. 3:**
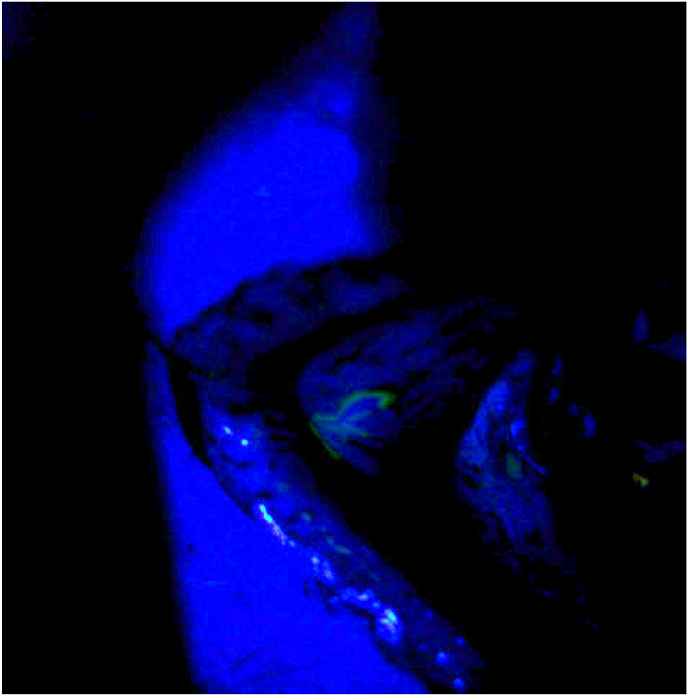
Fluorescein under cobalt blue light, demonstrating subtle flow at the conjunctival opening.

**Fig. 4:**
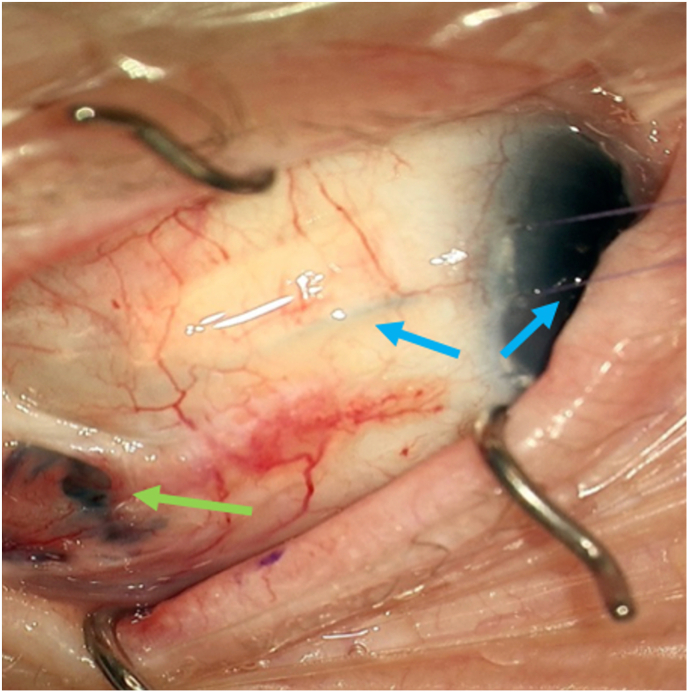
Trypan blue in anterior chamber traveling along glaucoma tube (blue arrows) and extravasating at area of suspicion for exposure (green arrow).

Intraoperatively, the suspected area of conjunctiva was visualized in proximity to the GDD (Fig. 1, Fig. 2). Next, 0.06 % trypan blue was injected through a paracentesis wound into the anterior chamber of the right eye. A brisk leak was observed over the anterior portion of the temporal aspect of the Ahmed plate and later found to be overlying the anterior plate edge (Fig. 3, Fig. 4). This finding indicated that the fluid leak origin was of intraocular source, and a result of GDD leakage from conjunctival fistula. The trypan blue dye was then rinsed out of the anterior chamber with balanced salt solution. The decision was made to explant the Ahmed valve and tube given the history of prior revision and the recurrent nature of the exposure. The plate and tube were removed, conjunctiva closed, and an Iridex P3 micropulse laser was utilized to achieve IOP reduction.

On post-operative day one, ophthalmic examination was notable for a BCVA of 20/400 and IOP of 17.5 mmHg in the right eye. Slit lamp examination demonstrated proper wound closure superotemporally with a now negative Seidel sign. On post-operative day ten, her BCVA remained 20/400, IOP 9.4 mmHg, and the wound remained Seidel negative.

## 3 Discussion

Glaucoma drainage devices are an important treatment option for refractory cases of glaucoma. While complication rates are relatively rare, GDD exposure is among the more serious complications of these surgeries, reported to occur in approximately 2.0 % of cases within five years post-operatively.^16^ Mechanical rubbing of the tube against the sclera as well as local inflammation that leads to scleral thinning have been proposed as possible etiologies for exposure, though other risk factors include female sex, older age, and prior intraocular surgeries.[17, [18, [19 In such cases, the timely diagnosis and management of exposed GDDs and their subsequent leakage is integral towards optimizing patients’ prognoses. The most serious consequences of GDD exposure include endophthalmitis and hypotony.^20^

Often the use of fluorescein dye to perform a Seidel test is adequate to identify a GDD exposure and leak. In rare circumstances, such as the patient in this report, complicating factors such as proximity to lacrimal gland and/or prior glaucoma and conjunctival surgeries may make it difficult to distinguish from pseudo-Seidel positivity. Unnecessary surgical revision of the GDD may result in additional surgical complications, and possibly the creation of a new defect from surgical manipulations. Alternatively, in the event of a true occult exposure, observation may risk progression to endophthalmitis. Given the importance of correct diagnosis, a more definitive diagnostic test is necessary.

In this case report, we outlined a novel technique that implements trypan blue dye to distinguish conjunctival fistulas secondary to GDD exposure from physiologic openings of the lacrimal gland, in other words, to distinguish true Seidel from pseudo-Seidel sign. Trypan blue has previously demonstrated efficacy in glaucoma surgeries, used to assess patency of trabeculectomy filtration blebs.^21^ During GDD repairs secondary to tube obstruction, trypan has also been applied to confirm GDD tube patency.^22^ We herein proposed a new technique highlighting trypan's efficacy in intraoperative diagnostics. The detection of stained leakage as observed in our case ultimately supports the diagnosis of a conjunctival fistula due to exposure. If there were no tube or plate exposure, the trypan dye would be expected to simply travel to the bleb overlying the plate and remain contained. There were no post-operative complications of trypan use in this case. However, at high concentrations, intracameral use of trypan blue has been associated with corneal endothelial cell toxicity, which may be of particular concern in eyes with GGDs.^23^ Other potential hazards of intracameral trypan include inflammation, infection, and in rare cases toxic anterior segment syndrome, in addition to the general risks of paracentesis including infection, inflammation, hypotony, and corneal and iris injury.^24^^,^^25^ These risks may be partially mitigated by ensuring short duration of trypan use during the case as well as thorough irrigation after the test is complete.

Our novel approach towards differentiating such fistulas from lacrimal gland duct openings also offers diagnostic insights that direct management. In our case, the decision was made to explant the GDD given our patient's history of prior exposure, difficulty closing a chronic fistula, and concern for high risk of future leaks. However, GDD exposure can also be managed with tube repositioning techniques, including placement into the ciliary sulcus or pars plana insertion, as well as tube coverage techniques involving corneal patch grafts, scleral patch grafts, and conjunctival flaps.^26^, ^27^, ^28^, ^29^ To reduce the risk of recurrent exposure after a conjunctival pedicled flap approach, care should be taken to ensure the flap is well vascularized (3:1 ratio of length to width), of adequate size to cover the exposure without excess tension, and control of dissection depth. Further technical considerations are outlined in the original description of this technique.^29^ By selectively staining the aqueous with trypan blue, this diagnostic technique encourages more accurate evaluations of suspected leaks. Primary limitations of this study include the sample size as well as the requirement of sterility to conduct the test. The need to make a paracentesis incision and inject the dye into the anterior chamber raises the possibility of surgical complications including infection.

Overall, this novel surgical use for injecting trypan dye into the anterior chamber is a safe and effective method for differentiating the underlying source of fluid flow in the vicinity of GDD. This technique can help definitively distinguish between pseudo-Seidel from true Seidel sign in cases where Seidel test alone yields an ambiguous result.

### 3.1 Patient consent

Written consent to publish this case has not been obtained. This report does not contain any personal identifying information.

## CRediT authorship contribution statement

**Hamidah Mahmud:** Writing – review & editing, Writing – original draft. **Yeabsira Mesfin:** Writing – review & editing, Writing – original draft. **Yingna Liu:** Writing – review & editing. **Yvonne Ou:** Writing – review & editing, Conceptualization. **Jonathan E. Lu:** Writing – review & editing, Conceptualization.

## Authorship

All authors attest that they meet the current ICMJE criteria for Authorship.

## Funding

This work was supported by the All May See Foundation and an unrestricted grant to the Department of Ophthalmology from Research to Prevent Blindness.

## Declaration of competing interest

The authors declare that they have no known competing financial interests or personal relationships that could have appeared to influence the work reported in this paper.
